# Can Allosteric Receptor-Protein Interactions in Receptor Complexes Be a Molecular Mechanism Involved in Cancer Immune Therapy?

**DOI:** 10.3389/fendo.2019.00574

**Published:** 2019-08-20

**Authors:** Dasiel O. Borroto-Escuela, Kjell Fuxe

**Affiliations:** ^1^Department of Neuroscience, Karolinska Institutet, Stockholm, Sweden; ^2^Department of Biomolecular Science, Section of Physiology, University of Urbino, Urbino, Italy; ^3^Grupo Bohío-Estudio, Observatorio Cubano de Neurociencias, Yaguajay, Cuba

**Keywords:** A2AR-TCR heteroreceptor complexes, allosteric receptor-receptor interactions, immunosuppression, T cell, cancer immunotherapy

## Abstract

Based on the work in the Central Nervous System with discoveries of allosteric receptor-receptor interactions in homo- and heteroreceptor complexes representing a major integrative mechanism in synapses and extrasynaptic regions, it is proposed that a similar mechanism may exist in the immunological synapses. We discuss a putative additional molecular mechanism for the ability of the inhibitory T cell signaling proteins CTLA-4 and PD-1 and the adenosine A2AR to diminish T cell activation leading to enhancement of cancer development. We suggest that in the same immunological synapse involving T cells and antigen presenting cells multiple heteroreceptor complexes may participate and be in balance with each other. Their composition can vary between functional states and among different types of T cells. The T cell receptor (TCR) and its accelerators, strongly enhancing T cell activation, can be under inhibitory control by T cell signaling proteins CTLA4 and PD-1 and also the adenosine A2AR through inhibitory allosteric receptor-receptor interactions in different types of heteroreceptor complexes. As a result, inhibitory tumor induced immunosuppression can develop due to a dominance of the inhibitory signaling causing a brake on the TCR and/or its accelerator and the cancer immunotherapy becomes blocked.

## Understanding the Mechanisms of Cancer Therapy Based on Protein Brakes on T Cells

The first phase of precision medicine in the treatment of cancer derived from the characterization of mutations that induced or inhibited intracellular signaling routes leading to the development of cancer (1). It produced a specific treatment of cancer in most patients having the mutation but with the drawback that the clinical responses were of limited duration. The second phase was produced by advancement of immunology at the molecular level which made it possible to improve our understanding of the complexity of the immune mechanisms. As a result, the impact of activation of T cells for cancer therapy was introduced.

Nobel laureates James Allison and Tasuku Honjo discovered cancer therapy by inhibition of negative immune regulation (2, 3). Their discoveries led to a new principle for immune therapy. Allison found that the T cell protein receptor CTLA-4 operates as a brake on T cell function which is activated by the T-cell receptor and the T-cell accelerator receptor, their combined activation leading to a strong immune response. Thus, there is a balance between inhibitory and activating T cell receptors regulating T cell function. Such a balance is observed also in the heteroreceptor complexes in the synapses and extrasynaptic regions in the CNS modulating synaptic function in brain circuits [see below and (4)]. Experiments performed by the Allison group suggested that altering this balance toward the activation of the T-cell function by removal of the inhibitory receptor signals may lead to a new cancer immunotherapy (5).

In parallel, the Honjo group discovered a protein (PD-1) located on the immune cells which they identified to be an immunoinhibitory receptor and acted as a brake on the stimulatory T cell immunoreceptors (3, 6).

This pioneering work by the two groups led to clinical trials based on the use of antibodies against the two immunoinhibitory receptors CTLA-4 and PD-1 which resulted in successful cancer immunotherapy, especially after PD-1 blockade, and combined blockade of the PD-1 and CTLA-4 (7, 8). It should also be noted that in our work in the brain we have proposed that cocaine addiction can be caused by cocaine induced formation of pathological A2AR-D2R-Sigma1R heteroreceptor complexes (9). They may represent long term memories involving a strong and permanent brake on D2R protomer signaling with A2AR protomer and the Sigma1R working together to produce the brake on D2R signaling and recognition (9).

Our theory on the molecular basis of learning and memory (9–12) may also be mentioned in view of its relevance for long-lived memory T-cells identified through increased expression of the interleukin 7 receptor (13). The molecular reorganization of the postsynaptic homo- and heteroreceptor complexes can produce a transient molecular engram that represents a short-term memory. The consolidation of the reorganized receptor complexes may lead to a permanent molecular engram. The consolidation can involve the transformation of parts of the receptor complexes into soluble molecules that can bind to the transcription factors and modulate their activity into forming adapter proteins that can consolidate the receptor panorama formed. It may maintain the new signaling.

**Figure 1:**
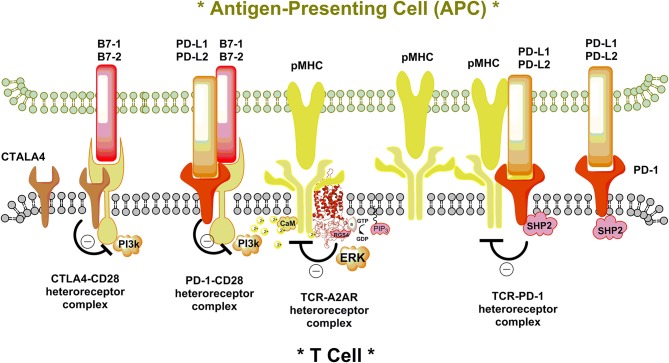
Illustration of a putative additional molecular mechanism for the ability of the inhibitory T cell signaling proteins CTLA-4 and PD-1 and the GPCR A2AR to diminish T cell activation leading to the enhancement of cancer development. The immune synapse between the antigen-presenting cell (APC) and T cell is shown. In the left part the accelerator costimulatory signaling protein CD28 in the plasma membrane of the T cell is shown being contacted by its activating protein B7-1linked to the plasma membrane of the APC. It is illustrated that that the inhibitory T cell proteins CTLA-4 and PD-1, activated by its ligands PD-L1 and PD-L2, may both (shown) or separately (not shown) directly contact CD28 to form a putative receptor complex. Through an inhibitory allosteric receptor-receptor interaction, the signaling of the CD28 becomes inhibited involving e.g., inhibition of phosphoinositide 3-kinase (PI3k) activation by CD28. To the right it is illustrated how the ligand bound PD1 may directly allosterically inhibit the T cell receptor (TCR) of the T cell, activated by the peptide bound major histocompatibility complex (pMHC). This may lead to reductions of TCR signaling through putative formation of a TCR-PD-1 complex. In addition, PD-1 L1 and L2 activation may also enhance the activity of protein tyrosine phosphatase 2 (SHP2) causing a reduction of phosphorylation of the TCR contributing to inhibition of TCR signaling. In the center of the immune synapse the adenosine activated adenosine receptor A2AR is proposed to directly interact with the TCR in the plasma membrane of the T cell to bring down its activation by pMHC through allosteric mechanisms. It is known that sustained activation of ERC produces strong T cell activation (15). It is therefore possible that in addition to the putative formation of TCR-A2AR heteroreceptor complexes, the A2AR induced activation of the AC-CREB pathway may also counteract the activation of the TCR by blocking sustained activation of ERK. This figure serves to indicate that the molecular mechanisms involve not only integration of the signaling pathways and their modulation of the signaling receptors involving changes in protein phosphorylation but also the allosteric integration in heteroreceptor complexes of the T cell through allosteric receptor-receptor and receptor-protein interactions. The balance of the heteroreceptor complexes with each other in the same T cell may also have a relevant role for the T cell function and help determine the state of the T cells.

In view of the above, it seems possible that integration in the T-cells not only involves changes in the signaling of the inhibitory and facilitatory immunoreceptors and integration of their intracellular pathways, but also their integration in immune synapses with the antigen presenting cells including the tumor cells. Based on the work in the CNS [see book edited by Fuxe and Borroto-Escuela (14)] it seems likely that integration in the immune system also involves the dynamic formation and operation of homo-and heteroreceptor complexes (Figure 1). The T cell receptor and the T cell accelerator receptors enhancing the immune response may form heteroreceptor complexes with the immunoinhibitory receptors CTLA4 and PD-1 inhibiting the immune responses in which the major integration of immune and transmitter/modulator signals take place (Figure 1). Multiple heteroreceptor complexes may be formed. The integration is proposed to be brought about through allosteric receptor-receptor and receptor-protein interactions which may be highly dynamic and dependent on the ligand concentrations for each receptor and the number of receptors and adapter proteins expressed in the immune synapses.

In parallel to the work above, the highly exciting work performed by Sitkovsky et al. (16–18) took place on the role of adenosine A2ARs in tumor-induced immunosuppression. It seems likely that also the A2AR can participate in some of these heteroreceptor complexes in the immune synapse and play an important role by favoring inhibition of the immune response (Figure 1). It will be of value to test if the A2AR antagonists may also enhance the immunotherapeutic actions of anti-CTLA4 and/or anti-PD-1 therapies.

## Integration of Signaling in the CNS Support the Above Hypothesis: Existence of Allosteric Receptor-Receptor Interactions in Synaptic and Extrasynaptic Heteroreceptor Complexes Including GPCRs

The Nobel Laureates Robert Lefkowitz and Brian Kobilka discovered the structure and function of the GPCRs (19). GPCRs of the different types were shown to form heteromers which means that they can physically interact with each other in the cytoplasm and/or in the plasma membrane (20–27). When the GPCRs are of the same type they are called homomers (20, 23, 28, 29). The term heteroreceptor complexes is used to describe that the receptor assemblies are built up of different receptors with unknown stoichiometry in combination with adaptor proteins. The latter can participate in the allosteric interactions between the receptors which can involve scaffolding functions to e.g., guide the receptors toward each other (9, 30, 31). The allosteric receptor-receptor interaction is a major mechanism in the heteroreceptor complexes. It develops when the binding of a ligand to an orthosteric or an allosteric site of one receptor protomer (present in an heteromer) via direct receptor-receptor interactions produces a change in another receptor protomer (present in the same heteromer) with regard to receptor binding, pharmacology, signaling, and/or trafficking (27, 32, 33).

The allosteric receptor-receptor and receptor-protein interactions result in highly diverse and biased signaling of the heteroreceptor complexes through changes in e.g., receptor recognition, G protein coupling involving switching between different G proteins. GPCRs interact not only with each other but also with ionotropic receptors (34, 35) and tyrosine kinase receptors (36–38). Reciprocal antagonistic or enhancing allosteric interactions can develop upon single or combined activation of the receptor protomers. The receptor interface of the various heteroreceptor complexes are currently being investigated and involve conserved amino acids that form hot spots that help bind the two interfaces together (39–41). Methods are presently being developed to determine the stoichiometry of the homo- and heteromers in cellular models and in tissues to know if we deal with dimers or higher order oligomers based on super-resolution and spatial intensity distribution methods (42–45). Early on higher order homo and heteromers were postulated to exist (46) and have now been demonstrated (47–49). The affinity of the receptors for each other and their relative amounts in the membrane domain studied will have a significant role in the heteromerization process.

Many of the homo and heteroreceptor complexes studied so far are mainly found in the extrasynaptic and synaptic regions of the plasma membrane of the neuronal networks using *in situ* Proximity Ligation Assay (PLA) and a neuronal marker antibody (14, 50). However, they can also exist in e.g., the astroglia based on PLA and astroglial markers like antibodies against glial fibrillary acidic protein. Furthermore, A2AR-D2R interactions in astroglia can modulate the gliotransmitter glutamate release from striatal astrocyte processes (51). Furthermore, cannabinoid receptors as well as the CB1-CB2 heteroreceptor complex have been demonstrated in activated microglia (52, 53).

These mechanisms in the brain are of particular interest for understanding the ability of A2AR antagonists or A2AR gene deletion to remove tumor-induced immunosuppression from tumor-reactive CD8^+^ T cells (17, 18).

## Possible Mechanism for the A2AR Mediated Brake on Immunosuppression of T Cells

Already in 1997 Sitkovsky et al. found that A2AR mediated signaling inhibited T cell activation (54). In 2012 the Sitkovsky group also obtained indications that the immunosuppressive functions of CD4 (+) CD25 (+) FoxP3 (+) regulated T cells involve A2AR signaling (16). Finally, in a highly significant paper in 2018 this group obtained evidence that tumor-reactive CD8(+) T cells can be liberated from tumor-induced immunosuppression by A2AR antagonists and by A2AR but not A2BR gene deletion (18). The hypoxia developed in the tumor microenvironment leads to increased expression of ectonucleotidases with enhanced formation of adenosine and activation of the A2AR involved in producing the immunosuppression. The A2AR may work together with the transcription factor HIF 1A, which is stabilized by hypoxia, to produce immunosuppression (18). The mechanism for the A2AR induced immunosuppression is proposed to be the increased formation of cAMP formed from the Gs coupled A2AR which activates intracellular pathways to inhibit the activation of the intracellular pathways of the T cell receptor (TCR). The existence of such a mechanism is supported by the ability of the A2AR antagonist to increase the extracellular levels of Interferon gamma. The release of pro-inflammatory cytokines is an important part of the signaling function of the T cell receptor in the T cells (18).

Based on our work in the brain as discussed above, another mechanism can also be involved, namely the formation of A2AR-TCR heteroreceptor complexes in the plasma membrane in which the A2AR can inhibit the function of the TCR through allosteric receptor-receptor interactions (Figure 1). It would be of high interest to determine if such receptor complexes exist in the immune system leading to inhibitory and/or facilitatory allosteric receptor-receptor interactions. This molecular mechanism represents a general integrative mechanism in the CNS and exist not only in nerve cells but also in glial cells (11, 55–57). Previously the focus was on integration in the intracellular signaling pathways from different receptors and is presently still the only integrative mechanism discussed in relation to communication in the immune system.

As discussed above, agonist induced activation of the A2AR protomer leads to a brake on D2R protomer signaling in A2AR-D2R complexes. Upon activation of the adaptor protein Sigma1R in this receptor complex, an enhancement of the D2R brake develops that may contribute to development of cocaine addiction (9). Higher order receptor complexes may therefore be considered to exist also in the immune system.

Finally, it may also be considered that A2AR-A2BR heteroreceptor complexes were demonstrated (58). In these complexes A2A receptor ligand recognition and signaling was blocked by A2B receptors. As a result, A2AR ligands may fail to act in T cells in which the A2AR-A2BR heteromer is the major population vs. the A2AR-TCR complex.

Based on the work of Sitkovsky and his group it can be of high relevance to test if also other GPCRs besides A2AR can be involved in tumor induced immunosuppression or enhancement. The chemokine receptor 5 (CCR5) is of interest as pointed out by one of the reviewers. It exists in the plasma membrane of T cells and can form an heterodimer with CXCR4 as shown with Fluorescence resonance energy transfer (FRET) (59). Chemokine-binding modulated this heterodimer and the CCR5 homodimer was specifically vulnerable to internalization by the protein partner Na+/H+ exchanger regulatory factor 1 (60). Thus, the type of CCR5 complex formed may determine the degree of CCR5 internalization obtained and thus its ability to mediate HIV transfection. CCR5 can also form heterodimers with CCR2b leading to negative binding cooperativity (61). These results illustrate the diversity of signaling that can develop with chemokine receptors through the formation of different types of receptor complexes. It may be proposed that distinct types of CCR complexes with a special protein composition may have the ability to form complexes that specifically can interact with TCR and/or accelerator receptors to reduce or enhance immunosuppression.

## Conclusions

The blockade by antibodies of the immunosuppressive proteins appears crucial for success in cancer immunotherapy (5, 7, 18). Based on the work in the CNS with discoveries of allosteric receptor-receptor interactions in homo- and heteroreceptor complexes representing a major integrative mechanism in synapses and extrasynaptic regions, it is proposed that a similar mechanism exists in the immunological synapses. In the same immunological synapse involving T cells and antigen presenting cells multiple heteroreceptor complexes can participate and be in balance with each other. Their composition can vary between functional states and among different types of T cells. It is illustrated that the T cell receptor (TCR) and its accelerators strongly enhancing T cell activation may be under inhibitory control by T cell signaling proteins CTLA4 and PD-1 and the GPCR A2AR through inhibitory allosteric receptor-receptor in different types of heteroreceptor complexes. However, higher order receptor complexes may also be formed where e.g., the inhibitory T cell signaling proteins CTLA4 and PD-1 can both participate in further enhancing the inhibition of the TCR and/or its accelerator (Figure 1). The same can be true for the A2AR when it is part of a higher order receptor complex in which also e.g., one of the inhibitory T cell signaling proteins participates. The cancer cells have the ability e.g., to increase the secretion of signals that activate the inhibitory T cell signaling proteins or the A2AR via increasing the levels of adenosine (18). As a result, inhibitory tumor induced immunosuppression develops due to a dominance of the inhibitory signaling causing a brake on the TCR and/or its accelerator. The removal of this brake has markedly improved immune cancer therapy (7).

## Data Availability

Publicly available datasets were analyzed in this study. This data can be found here: https://www.www.gpcr-hetnet.com.

## Author Contributions

We confirm and declare that all authors meet the criteria for authorship according to the ICMJE, including approval of the final manuscript, and they take public responsibility for the work and have full confidence in the accuracy and integrity of the work of other group authors. They have substantially contributed to the conception or design of the current review. Also they have participated in the acquisition, analysis, and interpretation of data for the current review version. They have also helped revising it critically for important intellectual content, and final approval of the version to be published. In addition, they have contributed in this last version of the manuscript in writing assistance, technical editing, and language editing.

### Conflict of Interest Statement

The authors declare that the research was conducted in the absence of any commercial or financial relationships that could be construed as a potential conflict of interest.
